# Popular Essays on Dentistry

**Published:** 1858-06

**Authors:** H. E. Peebles

**Affiliations:** St. Louis


### POPULAR ESSAYS ON DENTISTRY.

BY H. E. PEEBLES, D. D. S., ST. LOUIS.

It has ever been a maxim, with intelligent, honest men, pursuing the arts or sciences, that it is more pleasant and agreeable to deal with, as well as easier to satisfy, an enlightened and educated community, than an ignorant or illiterate one.

And in few cases, if any, is this maxim more pertinent than in the science and practice of Dental Surgery. For all operations, in the mouth and upon the teeth, as well as the mechanical appliances there, must ever be esteemed as only tolerable substitutes for diseased, deformed, or lost, natural parts, by all well informed persons. For owing to the peculiarity of the organs operated upon, or substituted, their importance or prominence in the animal economy, situated at the very head of the great alimentary canal, to serve as a receiving and comminuting apparatus, for all the materials that go to build up the tissues, and promote the functions of our system; to beautify and give expression to the human face divine, as well as the principal organs of speech.

But to the uninformed and ignorant, the operations of the dentist are expected to accomplish all, if not more, than nature does; and his appliances are expected to remedy fully the evil and inconvenience consequent upon the loss of the natural organs, and perform all and singular, the functions, at *least as well*, and be as comfortable as they ever were.

That our patients frequently say to us, "O, now sir, I shall never be troubled again with that tooth, as you have plugged it," or that they do not wish to have anything done to their "back teeth," for they want to let them all decay and come out, in order that they may have a new set made, and then they will have no more trouble with their teeth! is but too well known to our professional brethren.

And again, what proportion of the American mothers, know that there are only twenty teeth in the first or deciduous set, and thirty-two in the second or permanent set?

Now, in order to correct these errors, and remedy the evil consequent thereon, by instructing and enlightening their patrons, some of our professional brethren have published pamphlets, manuels, or hand-books, and circulated them among their patrons. But at the present day, and in the "Great West," this kind of *individual enterprise*, would be esteemed a species of puffing advertisement, savoring somewhat of charlatanism; hence no modest, honorable, high toned gentleman would venture upon the expedient alone, and yet we all feel and acknowledge that the people need light and instruction on these subjects.

Now who shall perform this work? Our authors do not, because they write for the profession, and not for the people.

This thing, it seems to me, can only be accomplished by circulating freely, generally, and frequently among the people, short, concise, and yet explicit papers, on each and all of the different operations and modes of treatment, as well as the mechanical fixtures usually resorted to in dentistry; not by individuals, but by societies and conventions in their associate capacities.

And these papers, or essays, thus going forth under the sanction, and name of respectable dental societies, will carry a weight, and be stamped with a dignity, that no one individual dentist can give them.

Then the people can be instructed; the profession vindicated, and all parties benefited.

